# Sensory neuropathy and nociception in rodent models of Parkinson's disease

**DOI:** 10.1242/dmm.039396

**Published:** 2019-06-27

**Authors:** Lucie Valek, Georg Auburger, Irmgard Tegeder

**Affiliations:** 1 Institute of Clinical Pharmacology, Goethe-University Hospital, 60590 Frankfurt, Germany; 2 Experimental Neurology, Goethe-University Hospital, 60590 Frankfurt, Germany

**Keywords:** Non-motor Parkinson's disease, Synuclein, Mitophagy, Mitogenesis, Protein aggregate, Pain, Sensory neuropathy

## Abstract

Parkinson's disease (PD) often manifests with prodromal pain and sensory losses whose etiologies are not well understood. Multiple genetic and toxicity-based rodent models of PD partly recapitulate the histopathology and motor function deficits. Although far less studied, there is some evidence that rodents, similar to humans, develop sensory manifestations of the disease, which may precede motor disturbances and help to elucidate the underlying mechanisms of PD-associated pain at the molecular and neuron circuit levels. The present Review summarizes nociception and other sensory functions in frequently used rodent PD models within the context of the complex phenotypes. In terms of mechanisms, it appears that the acute loss of dopaminergic neurons in systemic toxicity models (MPTP, rotenone) primarily causes nociceptive hyperexcitability, presumably owing to a loss of inhibitory control, whereas genetic models primarily result in a progressive loss of heat perception, reflecting sensory fiber neuropathies. At the molecular level, neither α-synuclein deposits alone nor failure of mitophagy alone appear to be strong enough to result in axonal or synaptic pathology of nociceptive neurons that manifest at the behavioral level, and peripheral sensory loss may mask central ‘pain’ in behavioral tests. Hence, allostatic combinations or additional challenges and novel behavioral assessments are needed to better evaluate PD-associated sensory neuropathies and pain in rodents.

## Abstract (precis)

**Summary:** Rodent models of Parkinson's disease partially develop prodromal somatosensory and olfactory dysfunctions reminiscent of sensory neuropathies in patients and reveal mechanistic insight, but data are incomplete and fragmented.

## Introduction

Parkinson's disease (PD) is a complex neurodegenerative disease that primarily affects motor systems of the basal ganglia (see Box 1 for a glossary of terms), but also multiple extranigral regions (Braak et al., 1995). The motor symptoms progress over time and include muscle rigidity, tremor, slowness of movement and difficulty walking (Darweesh et al., 2017). Dysphoric and anxious mood, depression and sleep disturbances also frequently occur, and PD-associated dementia manifests in some patients with advanced disease (Irwin et al., 2013). About 60-70% of patients with PD experience various types of acute or chronic pain, including neuropathic, visceral, musculoskeletal or dystonic pain, and headaches (Djaldetti et al., 2004; Djaldetti et al., 2007; Leclair-Visonneau et al., 2018; Yust-Katz et al., 2017; Zambito Marsala et al., 2011) often preceding motor impairments (Pont-Sunyer et al., 2015). The various types of PD pain have been well described and categorized (Beiske et al., 2009; Ford, 2010; Wasner and Deuschl, 2012), but the mechanisms are complex and still not completely understood.

**Box 1. Glossary**

**α-Synuclein (SNCA):** intrinsically disordered synaptic protein encoded by the gene *PARK1*. Mutations, including duplications, triplications and point mutations, increase its propensity for building SNCA aggregates and cause early-onset autosomal PD. The most frequent point mutations are A53T, A30P and E46K.

**Autonomous pacemaking:** autonomic generation of action potentials in neurons without an external stimulus, generating rhythmic activity.

**Autophagy:** cellular process to remove cellular waste such as misfolded or oxidized proteins, protein aggregates and damaged or dysfunctional organelles.

**Bacterial artificial chromosome (BAC):** DNA construct based on bacterial DNA that is used in transgenesis.

**Basal ganglia:** group of subcortical nuclei. The main components are the dorsal striatum (caudate nucleus and putamen; controls motor functions), ventral striatum (nucleus accumbens and olfactory tubercle), globus pallidus, ventral pallidum, substantia nigra and subthalamic nucleus.

**Ceramide, glucosylceramide:** sphingolipids essential for membrane structure and functions. Pathological levels of these molecules interfere with cell and organelle membrane functions. Metabolic intermediates for the generation of complex lipids such as sphingomyelin and gangliosides.

**Chaperone:** enzyme that catalyzes the folding of proteins.

**Chaperone-mediated autophagy:** cellular process to selectively remove long-lived cytosolic proteins. Target proteins are recognized via an exposed KFERQ-like motif and transferred to the lysosome by the chaperone Hsc70/Hspa8 and then directly imported via the lysosomal membrane receptor LAMP2a for degradation.

**Dysautonomia:** genetic or acquired dysfunctions of the autonomic nervous system with e.g. anhidrosis, constipation, tachycardia, bladder and orthostatic dysfunctions.

**Electro-oculogram** **(EOG):** electrodes are placed in the right and left outer ocular muscles to record changes of voltage between the cornea and retina during eye movement. Used for monitoring REM sleep.

**Electroolfactogram (EOG):** recording of electrical activity from the olfactory epithelium. Used in the diagnosis of anosmia.

**Enteric nervous system (ENS):** autonomic nervous system of the gastrointestinal tract.

**Hyperalgesia:** strong feeling of pain upon stimulation with a normally weakly painful stimulus.

**Hyperexcitability:** lowering of the excitation threshold or increased firing rate of a neuron.

**Induced pluripotent stem cells (iPSCs):** pluripotent stem cells that can be developed from somatic adult cells via reprogramming, which is achieved by transduction of a set of transcription factors. The original set consists of Oct4, Sox2, Myc and Klf4, referred to as Yamanaka factors after the first person to describe this technique.

**L-DOPA:** precursor of dopamine (DA); used for the treatment of PD.

**Lipopolysaccharide:** molecule of the outer membrane of Gram-negative bacteria. Activates Toll-like-receptors.

**Long-term potentiation (LTP):** form of synaptic plasticity with long-lasting enhancement of synaptic strength.

**Mitophagy:** removal of mitochondria via autophagy; requires labeling of mitochondria with ubiquitin.

**MPTP (1-methyl-4-phenyl-1,2,3,6-tetrahydropyridine):** neurotoxin precursor that is metabolized and thereby activated by monoamine oxidase B in the brain to MPP^+^, which preferentially destroys DA neurons because it is selectively imported via DA transporters.

**Multiple system atrophy (MSA):** rare neurodegenerative disease with autonomic dysfunctions, tremor, slow movement, muscle rigidity, postural instability and ataxia. MSA is a synucleinopathy (involving SNCA). Unlike in PD, SNCA pathology is mostly observed in glial cells. MSA is mostly unresponsive to L-DOPA. Transgenic phospholipid protein (*PLP*)-promoter driven SNCA expression in oligodendroglial cells is a model for MSA in mice.

**Nigrostriatal dopamine (DA) system:** DAergic pathway that connects the substantia nigra pars compacta with the dorsal striatum, i.e. putamen and caudate nucleus.

**Nucleus accumbens (NAc):** region in the basal forebrain involved in motivation, aversion, reward and reinforcement.

**Proteasome:** protein complex that degrades ubiquitin-labeled proteins one by one via proteolysis.

**Quantitative sensory testing (QST):** diagnostic battery of sensory tests to assess somatosensory functions in humans via determination of thresholds for cold and warm detection, cold and heat pain, paradoxical heat pain on cold stimulation, mechanical sensation and mechanical pain, vibration detection, and pressure pain. Similar tests are used in rodents to measure paw-withdrawal thresholds, reflecting sensory functions, including nociception.

**Rapid eye movement (REM) disorder:** sleep disorder (parasomnia), in which people act out their dreams. The normally occurring motor inhibition during REM sleep is lost and therefore allows movements.

**Restless legs syndrome:** a disorder that causes a strong urge to move the legs, particularly during rest and sleep. It occurs in the prodromal period of PD.

**Rotenone:** insecticide that blocks complex I of the mitochondrial respiratory chain; also blocks microtubule assembly.

**Small-fiber neuropathy:** peripheral neuropathy that results from damage to small unmyelinated peripheral nerve fiber terminals, categorized as C fibers and Aδ fibers. May affect the terminals of autonomic and somatosensory neurons including nociceptive neurons that innervate the skin and muscles (somatic fibers) and visceral organs (autonomic fibers). The complaints include paresthesias, dysesthesias, burning pain, visceral pain, constipation, bladder dysfunctions, dyshidrosis, salivation, etc. Diagnosis is based on QST and skin fiber density in skin biopsies.

**SNARE proteins:** protein complex that mediates docking and fusion of synaptic vesicles with the presynaptic membrane in neurons.

**Somatosensory cortex (SSC):** cortical region receiving input from the somatosensory system including the nociceptive system.

**Substantia nigra pars compacta (SNpc):** region in the midbrain with high neuromelanin content. Loss of DA neurons in the SNpc is a hallmark of PD.

**Ubiquitin-proteasome system (UPS):** cellular processes involving labeling of proteins with ubiquitin and subsequent protein degradation in the proteasome.

**Ventral tegmental area (VTA):** group of DA neurons on the floor of the midbrain involved in motivation, reward and reinforcement. VTA DA neurons are less vulnerable to PD pathology than SNpc neurons.

**Visual analog scale (VAS):** psychometric diagnostic tool for assessment of e.g. pain intensity. Subjects specify their level of agreement to a statement by indicating a position along a continuous line between two end points, e.g. ranging from no pain to maximum pain.

In addition to pain, other non-motor symptoms are highly prevalent in prodromal PD, particularly olfactory dysfunctions, rapid eye movement (REM) sleep disorder, dysautonomia and restless leg disorder (Box 1) (Berardelli et al., 2013; Postuma et al., 2012; Rana et al., 2013; Schrag et al., 2015; Snider et al., 1976). Particularly, chronic pain phenomena are often missed as early manifestations of PD, but are misdiagnosed and treated as low back pain, shoulder pain, sciatica, arthritis, depression etc. (Schrag et al., 2015). A few observational studies have shown that pain intensity ratings are associated with the severity of motor impairments and motor function scores (Allen et al., 2016; Vila-Cha et al., 2019), have a partial response to muscle-relaxing agents or cannabis (Balash et al., 2017; Lotan et al., 2014), and fluctuate in part with L-DOPA (Box 1) medication (Valkovic et al., 2015). PD pain is therefore often ascribed to muscle rigidity (Allen et al., 2016), but a recent large cohort study found that factors predicting overall pain included affective and autonomic symptoms, motor complications, female gender and younger age, but not motor impairment or disease duration (Silverdale et al., 2018).

Prodromal sensory manifestations of PD were first described about 40 years ago (Koller, 1984; Snider et al., 1976), and neurologists increasingly recognize that PD patients frequently develop small- or mixed-fiber sensory neuropathies (Box 1), which involve somatosensory and autonomic nerves (Kass-Iliyya et al., 2015; Nolano et al., 2008, 2017; Strobel et al., 2018; Zis et al., 2017). Patients frequently complain about constipation and other manifestations of autonomic nerve fiber damage or dysfunctions, including visceral discomfort and pain, gut and bladder dysfunctions, and deregulation of exocrine glands, whereas a loss of somatosensation is often unnoticed, unless it is associated with pain (Silverdale et al., 2018). Quantitative sensory testing (QST; Box 1) shows that about one third of PD patients develop progressive sensory neuropathies (Gierthmuhlen et al., 2009; Nolano et al., 2008, 2017) that not only cause polyneuropathic pain but are also known to contribute to musculoskeletal or widespread pain (Berglund et al., 2002; Kosek et al., 1996; Uceyler et al., 2018). In PD, this type of pain mostly does not respond to L-DOPA medication (Silverdale et al., 2018). Patients suffering from PD pain can be subdivided clinically into those with or without sensory neuropathy (Lin et al., 2016; Nandhagopal et al., 2010; Nolano et al., 2008), which may affect treatment options but so far has not provided mechanistic insight into this dichotomy. The progressive loss of sensory functions parallels the progression of the disease, but may precede motor symptoms for more than 10 years (Pont-Sunyer et al., 2015; Silverdale et al., 2018), suggesting that sensory neurons are particularly vulnerable to PD pathophysiology. The predominant phenotype consists of a loss of thermal perception, pathological heat pain and mechanical hypersensitivity (Gierthmuhlen et al., 2009; Nolano et al., 2008), and often co-occurs with a loss of olfactory sensitivity and discrimination (Devigili et al., 2008; Vollert et al., 2016). QST results in PD patients (Gierthmuhlen et al., 2010; Nolano et al., 2008; Strobel et al., 2018) mostly agree with a ‘denervation pattern’ type of sensory neuropathy, which is not unique to PD but also occurs, for example, in diabetes and other metabolic diseases (Vollert et al., 2016, 2017, 2015). Further sensory dysfunctions include visual problems owing to retinal pathology caused by α-synuclein (SNCA; Box 1) deposits in the retina (Jellinger, 2011; Ortuno-Lizaran et al., 2018) and alterations of taste preferences (Kashihara et al., 2011; Kim et al., 2011).

There are no apparent remedies to specifically alleviate PD-associated pain (Perez-Lloret et al., 2012; Sophie and Ford, 2012). Actually, the peripheral dopamine (DA) overload caused by treatment with L-DOPA is suggested to contribute to the development of sensory and autonomic neuropathies (Devigili et al., 2016; Nolano et al., 2017; Zis et al., 2017). For the majority of patients, there are no safer equally effective alternatives to L-DOPA, and preventive sensory-neuron-saving treatments are not available. Cannabis has been suggested as an effective treatment for painful sensory neuropathies (Abrams et al., 2007; Ellis et al., 2009) and often relieves pain in PD, likely not only due to its muscle-relaxant properties (Balash et al., 2017; Carroll et al., 2004; Lotan et al., 2014; Yust-Katz et al., 2017) but also direct analgesic and neuroprotective effects. Despite the progress in terms of availability of medical cannabis, pain in PD is still undertreated. To find additional or alternative effective treatments and methods for slowing the progression, researchers need to better define the pathophysiology in appropriate model organisms, including rodents and flies.

**Fig. 1.**
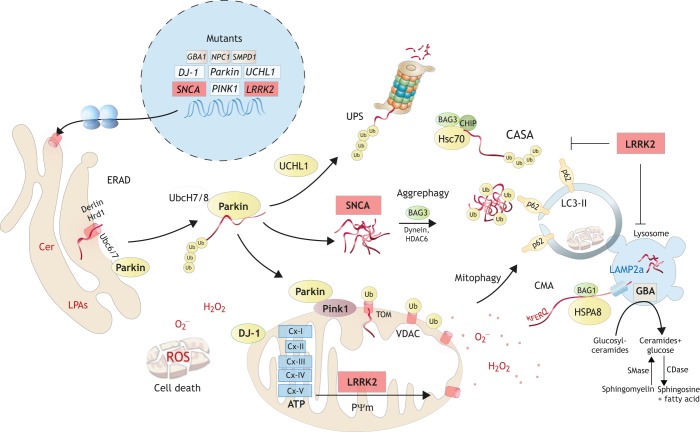
**Molecular functions of Parkinson's disease (PD) genes in rodents.** The pathoetiology of PD is characterized by disruption of mitochondrial integrity and protein allostasis, the latter owing to malfunctioning of the ubiquitin-proteasome system (UPS; Box 1) and of autophagy-mediated degradation pathways. Depending on the genetic cause, a combination of redox stress, energy shortage, protein aggregates and mitochondrial-DNA-evoked inflammation sets off a progressive accumulation of cellular waste ultimately resulting in cell death that manifests in a loss of vulnerable DAergic and sensory neurons. Mutations in α-synuclein (SNCA; encoded by *Park1*) or overexpression of wild-type SNCA disrupt the endoplasmic reticulum (ER)-to-Golgi transport by interfering with vesicle tethering and/or fusion. Similar events may disrupt synaptic vesicle release. SNCA is a constitutively unfolded protein and the mutant is prone to aggregation. It recruits other proteins to the aggregates and may overwhelm the aggresomes and ubiquitylated cargo proteins, all requiring functional E3 ubiquitin ligases including Parkin. Mutations in Parkin, UCHL1 and SNCA all interfere with the UPS, which may result in ER-stress responses leading to secondary inhibition of translation. Proteasome dysfunctions normally elicit alternative rescue mechanisms, including activation of chaperone-mediated autophagy (CMA), in which long-lived cytosolic proteins transfer to the lysosome via the LAMP2a receptor. However, mutant SNCA or gain-of-function mutants of LRRK2 cause CMA dysfunction. Mutant LRRK2 also contributes to mitochondrial pathology and increases mitophagy. The latter requires ubiquitylation of damaged mitochondria via Pink1/Parkin, in that depolarized mitochondria expose Pink1 on the outer membrane. Pink1 then recruits Parkin, which ubiquitylates outer-membrane proteins of the damaged mitochondrial fragment for autophagy-receptor-guided engulfment and mitophagy. LKKR2 is also a regulator of mitochondrial fission/fusion. Excessive fission is associated with mitochondrial dysfunction and increased ROS production. DJ-1 acts as a redox sensor and antioxidant in mitochondria, and helps to maintain energetic and redox homeostasis. Additional mutations associated with sporadic PD affect lysosomal enzymes, including glucosylceramidase β (GBA1) (Beavan et al., 2015; Murphy and Halliday, 2014; Robak et al., 2017), α-galactosidase A (GLA) (Alcalay et al., 2018; Wise et al., 2018; Wu et al., 2008), sphingomyelin phosphodiesterase 1 (SMPD1) (Wu et al., 2014) and Niemann Pick disease type 1 (NPC1). They all associate with lysosomal storage diseases, hence showing the genetic convergence of PD and lysosomal storage disorders and the importance of lysosomal pathways. Within the nucleus, white boxes show recessive PD genes, red boxes show dominant PD genes and gray boxes show PD-associated genes.

A number of rodent models have been developed, which have been extensively reviewed in the past (Chesselet et al., 2008; Creed and Goldberg, 2018; Duty and Jenner, 2011; Meredith and Rademacher, 2011; Terzioglu and Galter, 2008; Ubeda-Banon et al., 2012) and are mostly based on human genetics of PD (Alessi and Sammler, 2018; Kruger et al., 1998; Lucking et al., 2000; Polymeropoulos et al., 1997; Sidransky et al., 2009; Valente et al., 2004) (Table 1, Fig. 1). These models have been characterized in terms of motor behavior and PD-like histopathology, mostly with a focus on the basal ganglia. However, studies of non-motor manifestations investigating cognition, olfaction, anxiety-like behavior and gastrointestinal functions are fragmented and in part inconclusive. The present Review provides an overview of motor and non-motor behavior in popular rodent models of PD, with a focus on sensory phenomena and complex behavioral phenotypes, which may point to non-motor disease manifestations possibly including ‘pain’. We discuss whether a comparison of models and readouts can add to the current understanding of the mechanisms of somatosensory neuron vulnerability and dysfunctional pain signaling in PD.

**Table 1.**
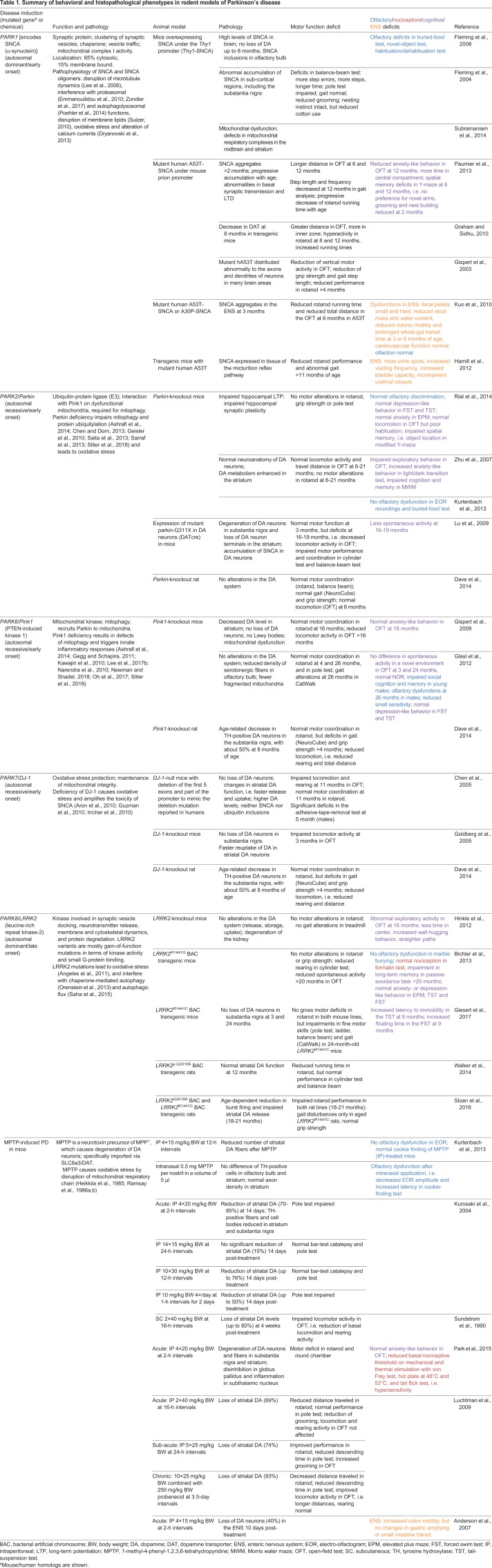
**Summary of behavioral and histopathological phenotypes in rodent models of Parkinson's disease**

## Nociceptive neurons in PD

The histopathology of PD is characterized by SNCA deposits in neuronal cell bodies (Lewy bodies) and neuronal processes (Lewy neurites), leading to a degeneration of the nigrostriatal DA system (Box 1), with neuronal loss and reactive gliosis in the substantia nigra (Box 1) (Dickson, 2012) and extranigral regions such as the amygdala, cerebral cortex, spinal cord, and brainstem areas that regulate autonomic functions (Braak et al., 1995; Braak et al., 2007; Del Tredici and Braak, 2012). Histopathological studies of mouse PD brains more or less recapitulate human histopathological features, although these are mostly more subtle (Gispert et al., 2003, 2009; Kurz et al., 2010) and involve regions that are normally not affected in humans. This suggests that rodents are less vulnerable and require add-on challenges or mutations to recapitulate profound ‘human-like’ morphological manifestations (Gispert et al., 2015; Sliter et al., 2018).

**Fig. 2.**
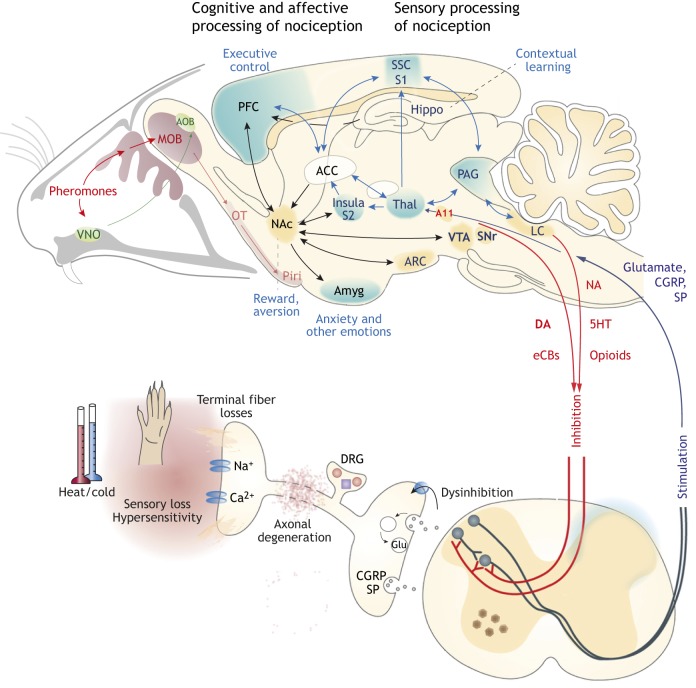
**Nociception and olfaction in PD.** Sensory processing of nociception involves primary nociceptive neurons in the dorsal root ganglia (DRG), secondary projection neurons in the dorsal horn of the spinal cord, the dorsolateral thalamus and somatosensory cortex (SSC, S1). This direct path connects to the prefrontal cortex (PFC), the insula cortex and the limbic system – amygdala (Amyg), anterior cingulate cortex (ACC), nucleus accumbens (NAc), areas of the midbrain [e.g. ventral tegmental area (VTA); periaqueductal gray (PAG)] and hippocampus. These areas process the cognitive and affective modulation of ‘pain’ and are needed to feel the reward associated with pain relief. This pain-relief reward is based on the release of DA in the NAc from VTA afferents and is strengthened by endocannabinoids. In addition, DAergic pain-inhibiting pathways arise from the midbrain and signal to the dorsal horn of the spinal cord. Although VTA neurons are less vulnerable to genetic causes or toxins than DA neurons of the substantia nigra, dysfunctions in these reward and pain-inhibitory pathways likely contribute to PD-associated pain. Sensory neurons are particularly vulnerable to defects of the ubiquitin-proteasome system (UPS), loss of mitochondria and inflammation, which result in axonal damage and loss of terminal nerve fiber endings. Clinically, fiber loss manifests as small-fiber or mixed-fiber sensory neuropathies, with sensory losses and pain. Rodent models of PD more or less recapitulate the sensory loss of smell, taste and nociception, which may precede motor-function deficits. Prodromal pain and olfactory deficits are highly prevalent, the latter resulting from degenerations of olfactory sensory neurons. SNCA deposits in the olfactory bulb spread to the projections to the olfactory cortex and areas involved in regulation of social behavior, nutrition and hormonal balances. AOB, accessory olfactory bulb; ARC, arcuate nucleus; CGRP, calcitonin-related peptide; eCBs, endocannabinoids; LC, locus coeruleus; MOB, main olfactory bulb; NA, noradrenaline; 5HT, serotonin; OT, olfactory tract; Piri, piriform cortex; SNr, substantia nigra; SP, substance P; Thal, thalamus; VNO, vomeronasal organ.

Evidence for an involvement of the pain matrix in PD patients comes from functional magnetic resonance imaging and positron emission tomography studies, and suggests that the somatosensory and insular cortex (Box 1, Fig. 2) and connectivity changes contribute to the misprocessing of pain in PD (Luo et al., 2014; Petschow et al., 2016; Polli et al., 2016; Tan et al., 2015). In mice, novel imaging techniques and *in vivo* recordings from the somatosensory cortex revealed a loss of dendritic spine density in a fibril seed model (Blumenstock et al., 2017) and loss of inhibitory interneuron activity in a neurotoxin-induced lesion model (Alam et al., 2017b), which would all agree with a hypersensitivity of the nociceptive system.

Immunohistochemistry of human spinal cord samples revealed SNCA deposits in lamina I neurons of the dorsal horn (Braak et al., 2007), which are pain projection neurons that receive input from peripheral nociceptive neurons and directly project to the thalamus and to sympathetic relay centers that modulate the parasympathetic regulation of the enteric nervous system (ENS; Box 1) (Braak et al., 2007). A more recent study described Lewy body pathology in neurons of the dorsal root ganglia and spinal cord in a large cohort of elderly subjects who underwent autopsy (Sumikura et al., 2015). From the relative amount, distribution and temporal occurrence of SNCA, the authors concluded that SNCA spreads from the nociceptive terminals in the dorsal horn to the somata in the dorsal root ganglia, and in the opposite direction from sympathetic ganglia to the intermediolateral column of the thoracic spinal cord (Sumikura et al., 2015). Hence, SNCA spreading may indeed originate in peripheral neurons.

Skin biopsies of PD patients revealed a high prevalence of small-fiber neuropathies or mixed-fiber polyneuropathies (Dabby et al., 2006; Donadio et al., 2014; Jellinger, 2011; Nolano et al., 2008; Wang et al., 2013), which agrees well with pathological QST results. The histopathology of skin biopsies or corneal confocal microscopy showed swollen axons and loss of terminal fiber density (Nolano et al., 2017; Podgorny et al., 2016), compensatory fiber sprouting (Nolano et al., 2017), and SNCA deposits in cutaneous sensory and autonomic nerves (Donadio et al., 2014; Wang et al., 2013). Similar alterations occur in rodent motor neurons in genetic and toxin-induced PD models (described in detail below). These were associated with signs of muscle dystrophy (Knaryan et al., 2011; Lee et al., 2002; Samantaray et al., 2008; Vivacqua et al., 2009), but the pathology of somatosensory neurons of the dorsal root ganglia or trigeminal ganglia has not been studied in rodents.

Nonetheless, PD rodents have provided insight into molecular mechanisms that likely apply to all neurons, although with substantial differences in their vulnerability. Very briefly, mitochondrial dysfunctions or defects of their axonal transport, complicated by failure of mitophagy (Box 1), cause oxidative stress and energy depletion (Ashrafi et al., 2014; Celardo et al., 2014; Chinta and Andersen, 2011; Koh and Chung, 2012; Ryan et al., 2013; Siddiqui et al., 2016; Dryanovski et al., 2013). The actions of the PD genes, PTEN induced kinase 1 (*PINK1*) and parkin RBR E3 ubiquitin protein ligase (*Parkin*; *PRKN*), which act in concert to target damaged mitochondria for removal via mitophagy, suggest that defective mitophagy is a clue to understanding the disease (Chen and Dorn, 2013; Geisler et al., 2010; Narendra et al., 2010). In addition, pathological SNCA imposes several toxic effects (Table 1), including disruptions of microtubule and membrane dynamics (Bendor et al., 2013; Bodner et al., 2010; Lee et al., 2006; Ludtmann et al., 2018), blocking of the proteasome (Zondler et al., 2017), and disruption of autophagolysosomal pathways of waste removal (Poehler et al., 2014), including autophagy, mitophagy and chaperone-mediated autophagy (Box 1) (Cuervo et al., 2004; Orenstein et al., 2013; Pan et al., 2008; Vives-Bauza et al., 2010; Wang et al., 2016). Consequently, susceptible neurons accumulate organelle, protein and lipid waste, and are unable to transport cargo to and from the synapse to the soma (Sulzer, 2010).

The selective vulnerability of DA neurons of the substantia nigra pars compacta (SNpc; Box 1) has been the focus of research (Burbulla et al., 2017; Guzman et al., 2010; Liss et al., 2005) leading to the concept that autonomous pacemaking (Box 1) in these neurons leads to sustained calcium influx through L-type calcium channels (Surmeier et al., 2011). Cytosolic calcium overload causes mitochondrial oxidative stress (Dryanovski et al., 2013) and subsequent redox modification of ATP-sensitive potassium channels (K-ATP) (Knowlton et al., 2018) that control the spontaneous tonic pacemaker activity (Liss et al., 2001, 2005; Schiemann et al., 2012), which may lead into a vicious cycle. Although nociceptive neurons are not spontaneously active, L-, N- and T-type voltage-gated calcium channels and K-ATP essentially contribute to the enhancement (calcium channels) (Alles et al., 2018; Candelas et al., 2019; Choi et al., 2016; Neugebauer et al., 1996) or lowering (K-ATP) of nociceptive neuron excitability (Kawano et al., 2009; Rodrigues and Duarte, 2000).

Aside the concept of calcium overload, owing to the long distances of axonal transport of organelles and vesicles, peripheral neurons are particularly vulnerable to alterations of microtubule dynamics. Autophagosomes are constantly built at the peripheral terminals of sensory nerves, and are transported towards the soma to remove synaptic protein waste (Maday and Holzbaur, 2012, 2014), which helps to fend off chronic pain in injury models (Altmann et al., 2016). Hence, sensory neurons, with their long axons, easily succumb to axonal transport defects, as exemplified by chemotherapy-induced neuropathies caused by microtubule-disrupting agents (Colvin, 2019; Zheng et al., 2012).

The recent association of PD with mutations of the lysosomal enzyme glucocerebrosidase (GBA1; Box 1) further suggests that lysosomal dysfunctions of lipid metabolism contribute to PD pain and sensory loss. GBA1 mutations increase SNCA toxicity (Kim et al., 2018; Suzuki et al., 2015) via accumulation of ceramide (Box 1) species, which also progressively accumulate in the olfactory bulb of *Pink1*-deficient mice (Torres-Odio et al., 2017) and likely further disrupt membrane dynamics and microdomains and, hence, neuronal excitability. Indeed, a strong link has been established between PD and lysosomal storage disorders (Aflaki et al., 2017; Migdalska-Richards et al., 2017; Murphy et al., 2014; Rocha et al., 2015; Stojkovska et al., 2018; Zunke et al., 2018), some of which are characterized by serious pain (Godel et al., 2017; Uceyler et al., 2011).

In addition to rodent neurons, sensory neurons have been successfully generated from human fibroblasts via induced pluripotent stem cell (Box 1) reprogramming (Eberhardt et al., 2015; Mis et al., 2019; Namer et al., 2019; Schrenk-Siemens et al., 2018; Wainger et al., 2015; Zimmer et al., 2018) and have opened a new avenue for research of sensory neuropathies in PD. However, sensory neurons can only model the peripheral aspect of PD pain, which results from a complex interplay of pathological sensory input and mal-processing in nociceptive circuits, including the basal ganglia (Chudler and Dong, 1995). Hence, rodent models remain valuable in understanding the complexity of PD pain.

## Rodent models of PD

Transgenic mouse and rat PD models are based on the complex nature of human PD genetics (Bonifati, 2014; Domingo and Klein, 2018) (Fig. 1, Table 1). These transgenic rodents express mutant human SNCA (Gispert et al., 2003; Graham and Sidhu, 2010; Paumier et al., 2013), leucine-rich repeat kinase 2 (LRRK2) (Giesert et al., 2017), Pink1 (Puschmann et al., 2017) or VPS35 (Chen et al., 2019; Ishizu et al., 2016). Alternatively, they are deficient in endogenous PD-associated genes, including *Pink1*/*PARK6* (Ferris et al., 2018; Gispert et al., 2009), *Parkin*/*PARK2*, *DJ-1*/*PARK7*, *LRRK2*/*PARK8* (Hinkle et al., 2012) or the lysosomal ATPase *ATP13a2*/*PARK9* (mouse/human homologs are shown) (Schultheis et al., 2013). These models more or less recapitulate the nigrostriatal pathology and clinical disease (Creed and Goldberg, 2018; Goldberg et al., 2003; Kelm-Nelson et al., 2018; Kitada et al., 2009; Rial et al., 2014), and have been reviewed elsewhere (Cenci and Ohlin, 2009; Creed and Goldberg, 2018; Duty and Jenner, 2011; Terzioglu and Galter, 2008). However, their predictive value for PD pain has not been systematically compared.

Recently, researchers introduced novel techniques to model PD via adeno-associated virus (AAV)-mediated mutant SNCA expression (Aldrin-Kirk et al., 2014; Delenclos et al., 2017; Eslamboli et al., 2007; Gaugler et al., 2012), SNCA fibril seed injections (Peelaerts et al., 2015; Peng et al., 2018; Volpicelli-Daley et al., 2011), and CRISPR/Cas9-mediated mutations even in non-human primates (Yang et al., 2019). However, most of these recent models have not been evaluated for sensory dysfunctions or nociceptive hypersensitivity (Peng et al., 2018; Yang et al., 2019). SNCA fibrils, with their prion-like properties, are particularly interesting because they cause a spreading dissemination reminiscent of human PD (Danzer et al., 2012; Loria et al., 2017; Rey et al., 2018, 2016). However, injected SNCA fibril seeds do not spread easily via peripheral nerves into the central nervous system (Manfredsson et al., 2018), and SNCA fibrils seeded into the sciatic nerve triggered little deposition of protein aggregates in the dorsal root ganglia compared to the ventral horn of the spinal cord (Ayers et al., 2018). Such fibril injections into the mouse sensory cortex trigger progressive protein aggregation and selective degeneration of Lewy-inclusion-bearing neurons, but the functional consequences for nociception have not been evaluated (Blumenstock et al., 2017; Osterberg et al., 2015). Similar spreading was observed upon seeding fibrils into the olfactory bulb (Bieri et al., 2018; Ubeda-Banon et al., 2014), which caused progressive olfactory dysfunctions and finally led to motor impairments (Rey et al., 2018, 2016). A further study found spreading to the retina after seeding the brain homogenate of old mutant SNCA A53T mice into the brain of young SNCA A53T mice (Mammadova et al., 2018). Researchers did not assess nociception in spreading models but, interestingly, intra-gastric administration of rotenone (Box 1) caused SNCA deposits not only in the ENS but also in the brainstem (Pan-Montojo et al., 2010), suggesting that SNCA spreading from the periphery reaches central pain-inhibitory centers and propagates the disease (Wakabayashi et al., 2010).

Further models rely on the local or systemic toxicity of 6-hydroxydopamine (6-OHDA), 1-methyl-4-phenyl-1,2,3,6-tetrahydropyridine (MPTP; Box 1) (Sedelis et al., 2001), rotenone or paraquate (Kaur et al., 2011; Testa et al., 2005). The toxicants are injected locally to induce circumscript nigrostriatal lesions or administered systemically to induce both nigral and extra-nigral lesions. Additionally, researchers use proteasome inhibitors, well known to cause sensory neuropathies (Alé et al., 2015; Carozzi et al., 2013), and nigrostriatal lipopolysaccharide (Box 1) injections to induce local degenerative and inflammatory processes that aggravate SNCA's toxicity (Castano et al., 2002; Choi et al., 2009; Lema Tomé et al., 2013). Topical models are valuable tools to address certain aspects of the pathoetiology of PD. However, they do not mimic the systemic nature of the disease and do not address well the slow progression from single defects to complex allostasis of mitochondrial and protein degradation pathways, which are reflected in an accumulation of transcriptional deregulations over time (Torres-Odio et al., 2017). To address the early occurrence of PD-associated sensory neuropathies, the present Review therefore focuses on genetic models and systemic MPTP- and rotenone-induced rodent PD models.

## Methods for testing motor and sensory functions in rodents

### Motor function

Sensorimotor tests are fundamental to assess varying degrees of nigrostriatal dysfunction and the efficacy of potential therapeutics (Fleming et al., 2004). The most common motor tests measure general activity and rearing in the open field, running performance on the rotarod, and coordination and velocity in the pole tests (for behavioral tests, see Table 2) (Sedelis et al., 2001). Although these tests are well validated, they often do not detect subtle alterations in the nigrostriatal DA system. For example, Parkin-deficient mice show normal rotarod running but fail in a challenging balance-beam test (Table 2) (Goldberg et al., 2003). Similarly, mice treated with moderate doses of MPTP master the rotarod but fail in an inverted-grid test (Table 2), which measures muscle strength (Tillerson et al., 2002). The open-field test shows general locomotion, exploration and rearing frequency either via automated recording or by video tracking (Bury and Pienaar, 2013). Grip strength is assessed by measuring the latency to fall spontaneously or upon tail-pulling from a hanging wire or mesh grid. The tests detect muscle or general weaknesses, whereas ataxia, tremor, balance and coordination are observed in balance-beam tests or with footprint or video-based analyses of the mouse gait (Brooks and Dunnett, 2009). Because sensory tests of nociception (Le Bars et al., 2001) rely on paw withdrawal or require locomotion, basic motor function tests are important for accurate interpretation of sensory test results. Basically, all behavioral tests are more or less influenced by motor functions.

**Table 2.**
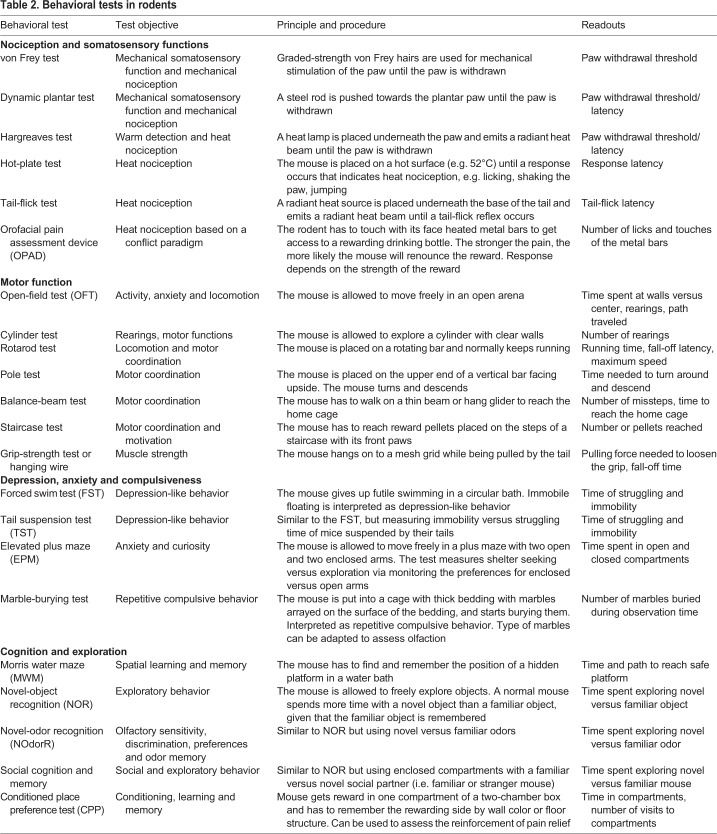
**Behavioral tests in rodents**

### Nociception

The most common tests of nociception apply heat, cold or mechanical stimuli to one or more paws and measure the withdrawal latency (Le Bars et al., 2001) (Fig. 2). The tests for thermal nociception include the hot-plate and cold-plate tests (Table 2) to determine paw licking, lifting or shaking latency, and the tail-flick and Hargreaves tests (Hargreaves et al., 1988) (Table 2), where a heat source is placed underneath the base of the tail or a hind paw to measure the latency of tail flick or paw withdrawal. Mechanical nociception is assessed as withdrawal threshold upon stimulation with von Frey hairs (Table 2) or with a thin steel rod with ascending force (dynamic plantar test; Table 2), or upon squeezing the paw (Le Bars et al., 2001). In addition, specific tests were developed for assessing headache or orofacial pain, including observation of spontaneous face rubbing upon injection of nitroglycerin (Akerman et al., 2013; Ferrari et al., 2016; Greco et al., 2018) and assessment of conflict behavior in the orofacial pain assessment device (OPAD; Table 2) (Cha et al., 2012; Hardt et al., 2019). Further nociceptive models rely on spinal reflexes evoked by injection of either formalin into a hind paw or acetic acid into the peritoneal cavity, causing quantifiable paw licking/flicking or abdominal writhing (Le Bars et al., 2001). These latter tests are still in use, but cause substantial animal suffering and are not useful for long-term observations of sensory functions in neurodegenerative models.

### Olfaction

Because loss of olfactory functions is a prodromal sign of PD in humans (Bohnen et al., 2010; Doty, 2012; Johansen et al., 2013; Postuma and Berg, 2016), researchers use olfaction tests to assess odor thresholds, preferences and social behavior in PD rodents (Table 2) (Fleming et al., 2008; Kurtenbach et al., 2013; Lehmkuhl et al., 2014; Ubeda-Banon et al., 2012) or flies (Chen et al., 2014; Poddighe et al., 2013). These tests rely on measuring the time or visit frequency to a pleasant odor, e.g. vanilla, offered in an open-field three-chamber box, radial maze or similar environment, or freezing behavior upon presentation of a predator odor. The use of mouse urine or a stranger versus familiar rodent as odorous stimuli additionally test social behavior. A normal mouse prefers the odor of a social partner over a neutral odor, and a novel over a familiar one (Lehmkuhl et al., 2014).

### Pain relief

Researchers need to consider that central processing of sensory input is influenced by the hedonic or anhedonic nature of a stimulus (Mrochen et al., 2016; Tinazzi et al., 2008), which is a function of DA signaling from the ventral tegmental area (VTA; Box 1) to the nucleus accumbens (Box 1), prefrontal cortex and amygdala (Lee et al., 2015; Peciña and Zubieta, 2015; Yu et al., 2014). Although VTA neurons are mostly spared or less affected in human PD (Liss et al., 2005; Neuhoff et al., 2002; Pacelli et al., 2015), dysfunctions within rewarding circuits likely contribute to the dysphoric mental status of PD patients and possibly PD rodents. Particularly, the rewarding feeling of pain relief and efficacy of placebos in humans involves DAergic signaling (Jensen et al., 2014; Loggia et al., 2014; Navratilova et al., 2015a; Navratilova and Porreca, 2014; Navratilova et al., 2012). Therefore, experience of reward or aversion may affect the readouts of nociceptive and olfactory tests in PD rodents. At the behavioral level, the conditioned place preference test (CPP; Table 2) measures how well a pleasant stimulus, e.g. relief of pain, is coupled with environmental features such as wallpaper and floor texture and is remembered in subsequent extinction periods (Navratilova et al., 2015b; Navratilova et al., 2012).

## Sensorimotor functions in rodent PD models

### Synuclein-based transgenic PD mice and rats

SNCA is a constitutively unfolded protein that regulates presynaptic vesicle release, possibly by functioning as a molecular chaperone of SNARE proteins (Box 1) (Chandra et al., 2005) or via interference with the recruitment of (phospho)lipids to SNARE complexes (Darios et al., 2010). Its toxicity mainly arises from SNCA oligomers and fibrils, which disrupt microtubules (Lee et al., 2006), damage mitochondria and increase oxidative stress (Abou-Sleiman et al., 2006), cause autophagolysosomal dysfunctions (Cuervo et al., 2004), inhibit the proteasome (Zondler et al., 2017), and alter membrane lipids (Suzuki et al., 2018). Hence, mutant SNCA, which has a high propensity for forming fibrils and aggregates, unites key mechanisms of PD that all contribute to the pathophysiology of sensory neuropathies. Considering the mechanisms of chemotherapy-induced or genetic sensory neuropathies (Zajaczkowska et al., 2019), any of these dysfunctions could result in sensory neuron loss with or without neuropathic pain.

SNCA duplications, triplications and point mutations cause rare familial autosomal-dominant early-onset PD. Five missense point mutations have been identified. Transgenic neuronal expression of the mutant human A53T variant (Polymeropoulos et al., 1997) is the best-studied SNCA-based genetic rodent model (Gispert et al., 2003; Graham and Sidhu, 2010; Kuo et al., 2010; Paumier et al., 2013; Recchia et al., 2008). Hence, sensory functions were mostly studied in SNCA A53T mutants (Fleming et al., 2004; Gispert et al., 2003) (Table 1). The other variants are A30P (Kruger et al., 1998), also used in genetic rodent and fly disease models (Chen et al., 2014; Recchia et al., 2008), E46K (Zarranz et al., 2004), H50Q (Appel-Cresswell et al., 2013) and G51D (Lesage et al., 2013), all resulting in strong SNCA oligomerization and gain of toxic functions. The A53T mutation and further SNCA variants also contribute to the risk for developing sporadic PD (Higuchi et al., 1998; Pavlou et al., 2017).

SNCA A53T animals replicate the human SNCA histopathology with Lewy bodies and Lewy neurites fairly well, but without loss of DA neurons and without spontaneous obvious PD-like motor-function deficits up until middle age. Instead, mice show subtle deficits of coordination and loss of fine motor skills, for example in balance-beam and pole tests (Fleming et al., 2004), whereas gait and rotarod tests were inconclusive (Fleming et al., 2004; Gispert et al., 2003). Reduced nest building and marble burying (Table 2) suggested deficits in olfactory functions or social behavior (Fleming et al., 2004; Gispert et al., 2003; Paumier et al., 2013). Indeed, compared to wild-type littermates, SNCA A53T mice detected and habituated to odors but with lower odor sensitivity and impaired discrimination, which was accompanied by SNCA inclusions throughout the olfactory bulb (Fleming et al., 2008). The involvement of the olfactory system was further confirmed in a histological study where tracing experiments revealed a loss of olfactory projections in homozygous SNCA A53T mice (Ubeda-Banon et al., 2012). Unfortunately, other sensory functions, including nociception, taste and visual functions, have not been assessed. Although hyposmia and pain frequently co-occur in PD patients (Baig et al., 2015), olfactory SNCA pathology does not predict an involvement of the somatosensory system (Sumikura et al., 2015). Notably, oligodendroglial expression of human naïve SNCA in phospholipid protein-SNCA mice, used as a model for multiple system atrophy (MSA; Box 1), caused very subtle changes in cold-plate responses, suggesting a loss of cold sensation. All other sensory tests in these mice were normal (Kuzdas-Wood et al., 2015).

### LRRK2/Park8 transgenic PD mice and rats

Autosomal-dominant gain-of-function missense mutations in *Park8*, which encodes LRRK2, account for 1-2% of all cases of PD and are the most common cause of familial PD. The frequency depends on ethnic background, with high prevalence in Ashkenazi Jews (15 to 20%) and North African Arab Berbers (up to 40%). LRRK2-associated PD closely resembles the symptomatology of idiopathic sporadic forms and has a late onset (Paisan-Ruiz et al., 2013), and some variations also increase the risk of sporadic PD (Domingo and Klein, 2018; Paisan-Ruiz et al., 2013). The penetrance of LRRK2 mutations is incomplete and age dependent, i.e. the G2019S variant is about 25% penetrant during life, R1441G about 95% penetrant at old age (Domingo and Klein, 2018; Lee et al., 2017a), which is important for interpreting the phenotypes of transgenic animals.

LRRK2 phosphorylates a subgroup of Rab GTPases and 14-3-3 proteins that regulate various cellular processes (Chan et al., 2011; Fujimoto et al., 2018; Lavalley et al., 2016; Stafa et al., 2014), including vesicular trafficking, autophagy and immune responses (Fig. 1). Pathogenic mutations mostly result in a gain of kinase activity that interferes with cytoskeletal dynamics and manifests in neurite retraction or shortening (Chan et al., 2011; Lavalley et al., 2016; Schwab and Ebert, 2015; Stafa et al., 2012), and would explain the losses of sensory nerve terminals. Cortical neurons from mutant LRRK2 knock-in rodents showed enhanced glutamatergic synaptic activity (Beccano-Kelly et al., 2014; Plowey et al., 2014), which would agree with nociceptive hypersensitivity.

LRRK2^R1441G^ bacterial artificial chromosome (BAC; Box 1) transgenic mice, which overexpress the R1441G mutation in the GTPase domain, showed mild hypokinesia in the open-field and subtle motor deficits in the cylinder test (Table 2) above 16 months of age, and gastrointestinal dysfunctions beginning at 6 months, pointing to prodromal autonomic nervous system dysfunctions (Bichler et al., 2013) (Table 1). However, LRRK2^R1441G^ mice performed normally in the accelerated rotarod test, and did not show a loss but only a functional deficit of DA signaling (Bichler et al., 2013). Non-motor features such as depression and anxiety-like behaviors in the elevated plus maze, forced swim and tail-suspension tests (Table 2) were all normal, and the mice had no cognitive deficits in learning and memory tasks. Nociception and olfaction in the formalin test and marble-burying tests were again all similar to littermate controls (Bichler et al., 2013). Hence, although the mice were somewhat slower at old age, they did not develop sensory deficits.

The R1441 site is a mutation hot spot, as the arginine can also be replaced by cysteine, serine or histidine (Mata et al., 2016; Ross et al., 2009), differently affecting the kinase activity. LRRK2^R1441C^ mice were compared to LRRK2 knockdown mice (Giesert et al., 2017), which theoretically should behave oppositely. Mice of both lines did not show gross motor deficits or DA neuron degeneration, neither at young (3-4 months) nor old (24 months) age. However, old homozygous LRRK2^R1441C^ mice had coordination deficits in the pole, ladder and balance-beam tests and catwalk gait analysis, and reduced olfactory sensitivity and discrimination, whereas LRRK2 knockdown mice were not affected in any of these tests (Giesert et al., 2017). Similarly, LRRK2 knockout mice with a deletion of exon 41 performed normally in motor tests, and DA storage, release, uptake and synthesis were not functionally compromised (Hinkle et al., 2012). Only exploratory activity assessed in the open-field test was subtly reduced, but importantly, the knockouts developed a kidney degeneration with progressive accumulation of autofluorescent material, which limits enthusiasm for LRRK2-inhibitor based treatments.

Because of the advances in BAC genetics and advantages of rats over mice (Creed and Goldberg, 2018), similar studies as above were performed more recently in transgenic rats carrying again the most frequent human LRRK2 mutations. Transgenic BAC rats expressing either the G2019S or R1441C variants were compared to wild-type LRRK2 animals up to 18-21 months of age (Sloan et al., 2016). Both mutants had L-DOPA-responsive motor dysfunctions, impaired striatal DA release, and cognitive deficits. In addition, *in vivo* recordings of SNpc DA neurons of R1441C mutant rats revealed a reduction of burst-firing rates but without overt neurodegeneration (Sloan et al., 2016). Unfortunately, sensory tests were not performed. Another study showed that rats overexpressing human LRRK2 G2019S had no abnormalities in positron emission tomography measures of striatal DA functions at 12 months (Walker et al., 2014), while performing subtly worse on the rotarod test, but not in the cylinder and balance-beam tests. Again, sensory functions were not assessed. Hence, the phenotypes of LRRK2 transgenic animals are subtle and diverse, but reflect the heterogeneous outcomes of LRRK2 variants in humans, and relatively large sample sizes will be needed for reliable assessment of sensory deficits.

### *Parkin*/*PARK2­*-, *Pink1*/*PARK6*- and *DJ-1*/*PARK7*-deficient PD rodents

The E3 ubiquitin ligase Parkin and the serine/threonine kinase Pink1 act in concert to maintain mitochondrial quality and to ensure efficient removal of damaged or dysfunctional mitochondria via mitophagy (Narendra et al., 2010; Sliter et al., 2018; Vives-Bauza et al., 2010). Healthy mitochondria actively import Pink1, direct it to the cristae membrane and rapidly degrade it (Beinlich et al., 2015; Fallaize et al., 2015). Upon mitochondrial depolarization, Pink1 translocates to the outer mitochondrial membrane (Beinlich et al., 2015; Fallaize et al., 2015) where it recruits Parkin, which, in turn, ubiquitylates damaged mitochondria and targets them for mitophagy (Fig. 1). Loss of Pink1 or Parkin function results in accumulation of damaged mitochondria, oxidative stress and cytosolic deposits of mitochondrial DNA (Sliter et al., 2018). The latter is recognized by DNA-sensing molecules and triggers innate immune responses (Sliter et al., 2018). The functions of the deglycase DJ-1 are less well understood. It cooperates with Pink1 and/or Parkin and promotes SNCA degradation via chaperone-mediated autophagy (Xu et al., 2017), and reduces mitochondrial oxidative stress induced by high cytosolic calcium (Guzman et al., 2010).

Loss-of-function mutations in the *Parkin*/*PARK2*, *Pink1*/*PARK6* and *DJ-1*/*PARK7* genes cause autosomal-recessive forms of early-onset PD. Parkin mutations are most common and account for up to half of clinical diagnoses of familial PD (Valente et al., 2004) and ∼15% of sporadic PD with a disease onset before the age of 45 years (Lucking et al., 2000). Mutations in *PARK6* and *PARK7* are less frequent, accounting for up to 1-8% and 1-2% of the sporadic cases with early onset, respectively (Bonifati, 2014; Kumazawa et al., 2008).

Single-knockout mice lacking one of these three recessive PD genes showed similar functional defects in the DA system, including reduced DA release probabilities from nigrostriatal terminals and impaired corticostriatal synaptic plasticity. However, the morphology and number of DA neurons in the SNpc were mostly normal up to 24 months of age (Goldberg et al., 2003, 2005; Kitada et al., 2007; Yamaguchi and Shen, 2007). Consequently, motor coordination of *Pink1*^−/−^ mice was normal in the rotarod test at 4, 16 and 26 months of age (Gispert et al., 2009; Glasl et al., 2012), and results in the pole test were inconclusive (Glasl et al., 2012; Kelm-Nelson et al., 2018). However, *Pink1*-deficient mice showed muscle rigidity and resting tremor leading to gait abnormalities and reduction of locomotion in the open-field test (Glasl et al., 2012). *Pink1* deficiency also impaired smell sensitivity (Gispert et al., 2009; Glasl et al., 2012; Kelm-Nelson et al., 2018) with a reduction of serotonergic fiber density in the glomerular layer of the olfactory bulb (Glasl et al., 2012) and progressive accumulation of ceramides and glucosylceramides (Box 1) in the olfactory bulb (Torres-Odio et al., 2017).

*Parkin*-knockout mice at 5-6 months performed normally in tests of olfactory discrimination, anxiety (elevated plus maze test), depression-like behavior (forced swimming and tail suspension) and motor functions (rotarod, grasping strength and pole tests) (Rial et al., 2014). However, they habituated badly to the open field and were slow or unable to locate objects, suggesting short-term spatial memory deficits (Rial et al., 2014). The behavioral phenotype was accompanied by impaired hippocampal long-term potentiation (Box 1), but long-term-potentiation-like phenomena in pain or olfactory circuits were not affected (Rial et al., 2014). Another study used electroolfactogram recordings (Box 1) to test olfaction in *Parkin*-knockout mice, again without overt differences (Kurtenbach et al., 2013). Reports concerning motor function in older mice (∼20 months) are contradictory, likely owing to differences in behavioral tests and setups, and include normal rotarod running (Zhu et al., 2007) but documented stepping errors in balance-beam tests and hypokinesia in open-field or cylinder environments (Lu et al., 2009).

Sensorimotor functions in *DJ-1*-knockout mice were investigated at 3 and 10 months of age. In young adulthood, mice showed reduced open-field locomotion (Chen et al., 2005; Goldberg et al., 2005) and, at middle age, reduced voluntary wheel running and rotarod fall-off latencies, likely owing to muscle stiffness or weaknesses caused by muscle fiber dysfunctions rather than loss of DA signaling (Zhou et al., 2017). Even *Parkin*/*DJ-1*/*Pink1* triple-knockout mice did not show overt histopathological features of PD. The morphology and numbers of DA and noradrenergic neurons in the substantia nigra and locus coeruleus were all normal up to the age of 24 months (Kitada et al., 2009), suggesting that, unlike humans, mice compensate a defect in one pathway (mitophagy) very well, at least in terms of gross motor functions and maintenance of DA neuron numbers.

In 2014, Dave and colleagues created and phenotyped the first single *Pink1*-, *DJ-1*- and *Parkin*-knockout rats, providing detailed insight into the pathology and behavioral characteristics of these animals (Dave et al., 2014). *Pink1*- and *DJ-1*-knockout rats both showed a progressive decline of tyrosine-hydroxylase-positive DA neurons in the substantia nigra, with a loss of about 50% at 8 months of age, accompanied by significant motor deficits starting at 4 months of age, including gait and strength abnormalities. Other motor deficits included reduced rearing behavior and total distances traveled in the open field, with a 70% decrease of mobility at 8 months but still normal rotarod running. The affected rats appeared to drag their hind limbs while ambulating. In contrast, *Parkin*-knockout rats were completely normal without any neurochemical or pathological changes up to old age (Dave et al., 2014). The researchers studied sensory or sensorimotor functions including startle responses, pupil response, response to touch and tail pinch, eye blink, and olfactory functions, but did not observe any significant differences for any of these parameters in any genotype at any of the three ages studied (4, 6 and 8 months) (Dave et al., 2014). Hence, rats recapitulate the human PARK6- and PARK7-associated motor function losses better than mice, but Parkin is apparently dispensable, and these knockout rats all do not show prodromal sensory impairments at least up to 8 months of age. Unfortunately, nociception was not investigated in these models.

### Systemic treatment with MPTP or rotenone

In addition to genetic PD models, neurotoxins are widely used to model PD as they induce local lesions in the substantia nigra via stereotaxic injection or to cause systemic toxicity by repeated intraperitoneal injections. MPTP, 6-OHDA (local only) and rotenone are the most versatile and best characterized. MPTP also causes PD in non-human primates (Chassain et al., 2001; Fox and Brotchie, 2010; Li et al., 2015; Potts et al., 2014; Seo et al., 2019) with some non-motor symptoms relevant to human PD, including a range of neuropsychiatric abnormalities and sleep disturbances (Belaid et al., 2015, 2014; Durand et al., 2015; Johnston and Fox, 2015). Nociception was not assessed.

Upon systemic injection, MPTP rapidly distributes to the brain, where it is metabolized into 1-methyl-4-phenyl-2,3-dihydropyridinium by the enzyme monoamine oxidase B and subsequently oxidized to 1-methyl-4-phenylpyridinium (MPP^+^), the active toxic compound. MPP^+^ is a structural DA analog and excellent substrate for the DA transporter (Slc6a3/DAT), explaining its selective toxicity for DA neurons (Sian et al., 1999). MPP^+^ blocks complex I of the respiratory chain, resulting in abrogation of ATP synthesis, accumulation of reactive oxygen species and, finally, mitochondrial dysfunction and cell death (Nicklas et al., 1985; Ramsay et al., 1991, 1986b).

In systemic-toxicity rodent PD models, MPTP is repeatedly injected intraperitoneally over a couple of days, causing a loss of DA neurons in the substantia nigra (Table 1). Single intraperitoneal injections of low doses of MPTP (e.g. 0.1 and 2 mg/kg body weight) only decrease the expression of tyrosine hydroxylase without causing a loss of DA neurons (Alam et al., 2017a). Although the MPTP model is broadly used and motor functions have been extensively studied (Table 1) (Kurosaki et al., 2004; Kurtenbach et al., 2013; Park et al., 2015; Sedelis et al., 2001; Sundstrom et al., 1990), there are only a few reports addressing olfaction (Kurtenbach et al., 2013) or nociception (Park et al., 2015). In the latter, injections of 4×20 mg/kg body weight at 2 h intervals resulted in a drop of paw withdrawal thresholds upon thermal (tail flick) and mechanical (von Frey) stimulation. The tail-flick hypersensitivity was reversed upon L-DOPA treatment (Park et al., 2015), but thresholds were measured only once after MPTP, although long-term effects and sensory neuron pathology were not assessed and inflammatory markers in the spinal cord not affected. Considering DAT-mediated selective import of MPP^+^ into DA neurons, the authors concluded that MPTP initially destroys descending inhibitory DA neurons, which arise from a small group of DA neurons in the midbrain, and that the hypersensitivity was caused by a loss of inhibition (Park et al., 2015). However, the results would also be compatible with acute direct toxic effects on nociceptive neurons resulting in hyperexcitability (Box 1), similar to the acute toxic effects of e.g. streptozotocin used to induce diabetes. Nociceptive hypersensitivity was also observed after local bilateral injection of 6-OHDA into the SNpc, which resulted in a loss of inhibitory medullar 5-hydroxytryptophan-positive neurons and their descending fibers that terminate in the dorsal horn of the spinal cord. The hyperalgesia (Box 1) was therefore again attributed to a loss of inhibitory pain control (Wang et al., 2017). Studies of olfaction showed normal electroolfactory recordings and cookie-finding times after intraperitoneal MPTP (Kurtenbach et al., 2013), but intranasal MPTP had local toxic effects on the olfactory epithelium, which manifested as lowered olfactogram amplitudes (Kurtenbach et al., 2013). Similar toxic effects occurred with intranasal rotenone (Sasajima et al., 2015).

Rotenone is a lipophilic insecticide that easily distributes to the brain. Like MPP^+^, it is an inhibitor of complex I of the mitochondrial respiratory chain, hence leading to formation and accumulation of reactive oxygen species, loss of ATP generation and mitochondrial damage (Fato et al., 2009). The rotenone model of PD has caught some attention since Betarbet et al. (Betarbet et al., 2000) first developed a PD model based on continuous intravenous infusion of low-dose rotenone (3 mg/kg body weight per day) for 33 days in Lewis rats. The animals developed motor deficits and nigrostriatal pathology reminiscent of human PD, with the value of the model being confirmed in subsequent studies (Cannon et al., 2009; Johnson and Bobrovskaya, 2015). Interestingly, repeated intra-gastric doses of rotenone did not only evoke SNCA deposits locally in the ENS, but also in the vagus nucleus (Pan-Montojo et al., 2010), suggesting that rotenone treatment models PD-associated dysfunctions of the autonomous nervous system, possibly visceral pain and the peripheral-to-central spreading of SNCA. Two studies presented evidence that rotenone can induce a loss of myenteric neurons of the small intestine (Drolet et al., 2009), associated with a delay in gastric emptying (Greene et al., 2009). Chronic rotenone administration also resulted in changes in cognitive functions (Kaur et al., 2011) and increased slow-wave and REM sleep during active periods (Yi et al., 2007). The excessive sleepiness was attenuated by intracerebroventricular administration of an interleukin-1-β receptor antagonist, but not with DA or a γ-aminobutyric acid (GABA) antagonist (Yi et al., 2007), suggesting that PD-associated REM sleep disorders may be exacerbated by an inflammatory component. Rotenone-induced sleep disturbances resemble the sleep disorder of SNCA A53T mutant mice, which showed increased REM sleep and a shift in the electroencephalogram power density toward lower frequencies during active times (McDowell et al., 2014). Hence, rotenone treatment mimics several non-motor manifestations and its variable dosing schedules makes it a flexible approach for modelling. However, killing sensory neurons with an insecticide will likely not unravel the biology of PD pain.

## Conclusions and future challenges

Owing to advances in transgenic technologies, researchers introduced valuable rodent models of PD in recent years, and whole-genome sequencing studies (Chang et al., 2017) promise further PD models in rodents and flies in the near future. Nevertheless, the data on nociception remains sparse, reflecting the difficulty to model and assess PD-associated pain in rodents because of its complex behavioral manifestations and equally complex etiology at the molecular and synaptic network levels. Even the same mutation may produce different phenotypes based on the animal's genetic background, sex, age and experimental settings.

In addition, it is particularly difficult to study nociception in the presence of peripheral sensory neuropathies with sensory losses, because the readouts (Table 2), mostly paw withdrawal, rely on perception of the stimulus and sensorimotor coupling. Even in humans, QST results often do not agree with pain intensity ratings revealed with visual analog scales (Box 1) or questionnaires (Djaldetti et al., 2004). Hence, although many studies included somatosensory tests and revealed a loss of thermal and/or mechanical perception, there is hardly any information about nociceptive hypersensitivity and spontaneous nociception, and model-inherent motor impairments preclude the performance of paw-withdrawal-based nociceptive experiments after the onset of serious motor impairments. Indirect readouts suggesting ‘pain’, such as higher frequencies of awakenings during sleep or loss of exploratory behavior, are influenced by non-pain-related dysfunctions or require locomotion and cognition. Hence, major future challenges will be to design studies that assess behavioral observations in combination with physiological *in vivo* readouts in home cages in social groups over extended periods.

Based on the current literature, one may infer that nociceptive hypersensitivity predominates upon acute toxin-mediated destruction of DA neurons owing to a disruption of inhibitory circuits, whereas thermal or mechanical insensitivities predominate in slowly progressing genetic models owing to direct axonal damage and loss of somatosensory nerve terminals. However, early or concomitant ‘pain’, which frequently accompanies sensory neuropathies in humans (Huge et al., 2008; Lang et al., 2006; Uceyler et al., 2018), might have been missed in many studies because visual-analog-scale-based pain intensity ratings and questionnaires are not available for rodents, and scorings according to the mouse grimace scale are useful for assessment of acute ‘pain’ such as postoperative pain (Langford et al., 2010) but not for slowly progressive neurodegenerative diseases. In comparing the models and considering the specific functions of the mutant genes, it appears that models with substantial mitochondrial damage combined with transport deficits mimic PD-associated sensory phenomena better than those that rely primarily on protein mal-disposal and aggregation. At the molecular level, neither SNCA deposits alone nor mitophagy failure alone appear to be strong enough to result in axonal or synaptic pathology of nociceptive neurons that manifest at the behavioral level, which is not unique for PD models. Although the slowly progressing genetic PD models appear to be somewhat disappointing for the researchers who seek robust models with strong phenotypes, low variability and timely onset, these models recapitulate the human disease, heterogeneity and age dependency well.

Like in humans, sensory phenomena in PD rodents precede motor dysfunctions as far as determined, and aging is an essential cofactor. Sensory phenotypes described in PD patients bear some similarity with the sensory neuropathies caused by metabolic diseases such as diabetes (Vollert et al., 2018, 2017) and mostly follow a ‘denervation phenotype’, suggesting that mitochondrial pathology and axonal transport deficits, possibly also involving mitochondrial-DNA-evoked inflammatory processes, are key determinants for PD-associated sensory neuropathies and pain. Studies of sensory functions in genetic PD models may therefore point at general mechanisms underlying the vulnerability of sensory neurons and in turn suggest that PD patients may benefit from e.g. chaperones or T-type calcium channel blockers considered as treatments for pain in sensory neuropathies. However, the limitations of behavioral tests need to be solved to reliably assess nociception in PD rodents, which will be essential to develop and test putative progression-slowing treatments in the future.
